# Mean and variability in functional brain activations differentially predict executive function in older adults: an investigation employing functional near-infrared spectroscopy

**DOI:** 10.1117/1.NPh.5.1.011013

**Published:** 2017-09-26

**Authors:** Drew W. R. Halliday, Bryce P. Mulligan, Douglas D. Garrett, Stefan Schmidt, Sandra R. Hundza, Mauricio A. Garcia-Barrera, Robert S. Stawski, Stuart W. S. MacDonald

**Affiliations:** a University of Victoria, Department of Psychology, Victoria, British Columbia, Canada; b University of Victoria, Institute on Aging and Lifelong Health, Victoria, British Columbia, Canada; c Max Planck Institute for Human Development, Max Planck UCL Centre for Computational Psychiatry and Ageing Research; Center for Lifespan Psychology, Berlin, Germany; d University of Victoria, School of Exercise Science, Physical and Health Education, Victoria, British Columbia, Canada; e Oregon State University, School of Social and Behavioral Health Sciences, Corvallis, Oregon, United States

**Keywords:** neural variability, executive functioning, older adults, standard deviation

## Abstract

Objective: although the preponderance of research on functional brain activity investigates mean group differences, mounting evidence suggests that variability in neural activity is beneficial for optimal central nervous system (CNS) function. Independent of mean signal estimates, recent findings have shown that neural variability diminishes with age and is positively associated with cognitive performance, underscoring its adaptive nature. The present investigation sought to employ functional near infrared spectroscopy (fNIRS) to derive two operationalizations of cerebral oxygenation, representing mean and variability [using standard deviation (SD)] in neural activity, and to specifically contrast these mean- and SD-oxyhemoglobin (HbO) estimates as predictors of cognitive function. Method: a total of 25 older adults (71 to 81 years of age) completed a test of cognitive interference (Multisource Interference Task) while undergoing fNIRS recording using a multichannel continuous-wave optical imaging system (TechEn CW6) over bilateral prefrontal cortex (PFC). Time-varying covariation models were employed to simultaneously estimate the within- and between-person effects of cerebral oxygenation on behavioral performance fluctuations. Results: mean effects were predominantly observed at the between-person level and suggest that greater concentrations of HbO are associated with slower and less accurate performance. Greater HbO variability at the between-person level was associated with slower performance, but was associated with faster performance at the within-person level. Conclusions: these findings are in keeping with assertions that mean and variability confer complementary (as opposed to redundant) sources of information regarding the effective functioning of a neural system and suggest that fNIRS is a viable methodology for capturing meaningful variance in the hemodynamic response that is characteristic of adaptive CNS function.

## 1 Introduction

Moment-to-moment variability in neural activity is an emerging area of research that shows promise for elucidating nuances of the human nervous system. In stark contrast to the number of studies examining cognitive behavioral and physiological variability, there is a paucity of research on variability in neural activity, despite the longstanding knowledge that neural variability is not merely noise, but rather a central feature of a stable and well-functioning neural system.^1^^–^^3^ In the present context, neural variability refers to within-person fluctuations in functional brain activity, with evidence to date primarily derived from functional magnetic resonance imaging (fMRI) and electroencephalogram (EEG) research. Neural variability exhibits an inverted U-shaped pattern, increasing through early life and declining through late life, with higher levels generally considered adaptive.^2^^,^^3^ Theoretically, increased neural variability is indicative of a more sophisticated neural system that can explore multiple functional states.^3^ Variability in neural activity may be functionally significant by facilitating a greater dynamic range of potential responses, according to Bayesian optimization and by enabling itinerant dynamics to avoid determinacy. Indeed, variability in brain activations may arguably represent the substrate for adaptive and stable neural function.^3^

Empirical support for the functional significance of neural variability includes examples from lifespan developmental phenomena as well as linkages to behavioral performance. McDonnell and Ward^4^ argue that neural networks are more robust when they are generated in the presence of greater noise (through “stochastic facilitation”), which is further supported by studies showing increased neural variability from infancy to early adulthood.^5^^–^^8^ In contrast, decreased variability in the functional magnetic resonance imaging blood-oxygen-level dependent (fMRI-BOLD) signal has been associated with increasing age and diminished behavioral performance in older adulthood.^3^^,^^9^^,^^10^ Recent investigations of variability in cerebral oxygenation, using either functional near-infrared spectroscopy (fNIRS) or fMRI, have shown positive associations with superior behavioral performance during measures of scene recognition^11^ and cognitive flexibility, but not cognitive stability,^12^ suggesting that neural variability during higher-order cognitive tasks is not only beneficial, but also construct specific. Further, Garrett and colleagues^13^ examined the impact of increasing cognitive demands on the modulation of brain signal variability. Increasing cognitive load was associated with broad (i.e., multiregion) increases in brain variability for younger and faster-performing adults, but comparatively fewer changes in brain variability for older and slower-performing adults. These age group differences, particularly under increasing cognitive load, were interpreted to reflect a neural system for the younger adults characterized by greater dynamic range between brain states (fixation versus cognitive demand) and an enhanced ability to efficiently process stimuli.

Neural variability also appears to be sensitive to developmental phenomena at both ends of the lifespan and to recovery following injury. EEG data from infants^5^^–^^8^ and fMRI data from older adult populations^3^^,^^9^^,^^10^ suggest a developmental trajectory of increasing-then-declining neural variability. Garrett and colleagues^9^ observed that older adults exhibited less neural variability than their younger counterparts, and direct comparison of standard deviation-versus mean-based BOLD patterns indicated that the former shared a five-fold stronger association with age. Further, increased neural variability has been associated with superior behavioral performance^3^^,^^6^^,^^8^^–^^10^^,^^12^^,^^13^ and better recovery from traumatic brain injury (TBI).^14^ The majority of studies examining neural variability in older adults have used fMRI to index the BOLD signal; however, given that the average sampling rate of fMRI methodology is comparatively slow (e.g., Garrett and colleagues^3^^,^^9^^,^^10^ used a repetition time (TR) of 2000 ms, resulting in 1 image every 2 s), these studies provide a relatively coarse estimate of BOLD variability reflecting sample-to-sample fluctuations. Further investigation of variability in cerebral oxygenation is therefore an important avenue of exploration, and may be better suited to neuroimaging methodologies indexing cerebral oxygenation that use faster sampling rates (e.g., fNIRS with signal sampling occurring once every 20 ms), to derive estimates that are statistically more precise. For example, for a single 30-s performance block, the SD estimate for a common fMRI TR of 2 s would be based upon only 15 samples (one every 2 s); in contrast, for the same length block within an fNIRS paradigm (at 50 Hz sampling), the within-person SD would be computed across 1500 individual samples (50  per second×30  s). As the hemodynamic signal is inherently noisy,^15^ and more importantly as moment-to-moment brain signal variability is hypothesized to also contain a durable characteristic signal independent of stochastic noise,^1^^,^^3^^,^^16^ the increased sampling density of fNIRS may be particularly useful for deriving precise and reliable estimates of neural variability.

### 1.1 Within- and Between-Person Analyses

Given that the majority of the extant neural variability literature is based on average between-person effects (e.g., does the older age group exhibit less variability than the younger age group?), it is of particular interest to investigate whether associations between behavior and neural function are also observed within persons (i.e., for any given individual, on occasions when neural variability is higher, is behavioral performance also better?). It has long been known that analyses executed at separate levels of nested-data hierarchies (i.e., the between- and within-person levels) do not necessarily yield equivalent results. Referred to as the ecological fallacy,^17^ results at the individual level may be of particular magnitude and direction, but when aggregated at the group level, can not only differ in pattern but may also be influenced by between-person confounds (e.g., age and cognitive status) that may obscure the effect of interest. For example, it is both conceptually and statistically possible for predictors to account for large proportions of variance within-persons, but to exhibit relatively little or no effect (or even opposite effects) when pooled across individuals.^18^ Similarly, research on ergodicity^19^ underscores the importance of examining within-person associations and suggests that (a) process-oriented phenomena (such as neural variability) are best examined within-persons over time and (b) a singular reliance on between-person averages to study such associations is incongruent with theoretical accounts. Therefore, despite being defined by the same outcome, the variation at between- and within-person levels of analysis may represent and be driven by completely different theoretical constructs (e.g., the reason person A performs better on a cognitive task relative to person B may be completely different from why both individuals each exhibit gains across the duration of a task).

### 1.2 Present Study

In the present study, we examined mean and variability operationalizations of the cortical hemodynamic response, at both the within- and between-person levels, for older adults performing an executive functioning task. Several key research aims were explored. First, functional estimates for mean and variability during an executive function task were derived. Relative to fMRI, the faster fNIRS sampling frequency arguably yields a more precise and reliable estimate of neural variability. Although several operationalizations of neural variability have been employed including a block normalized SD approach (fMRI literature) as well as multivariate multiscale entropy (MSE: EEG literature), these various approaches and the signals they index may not be equivalent.^3^ The findings reported in this investigation are based upon the normalized SD computation.^9^

Time-varying covariation models were then employed to estimate the within-person (at the individual level) and between-person (at the sample average level) associations for these two oxyhemoglobin (HbO) indicators on behavioral cognitive performance [Multisource Interference Task (MSIT) response latency and accuracy] across all performance blocks within the experiment. For variables such as functional activation that are studied over any longitudinal interval, an estimate for any given cross-sectional sample (e.g., block 1) comprises both between- and within-person sources of variance.^20^ Accordingly, failure to decompose variance into between- and within-person sources results in estimates that conflate the two—of particular concern to the extent that between-person variance for the phenomenon under study (e.g., the association between neural variability on cognitive performance) is distinct from patterns observed for within-person variance. Our investigation is particularly innovative as most investigations of neural variability are based upon between-person differences; here, we directly examine effects at both the between-person (i.e., effects that pertain to differences in level of neural variability between persons in relation to cognitive function) and within-person (i.e., effects that pertain to neural variability and how this couples with cognitive function within an individual) levels. As so few fMRI and fNIRS investigations have explored neural variability, with virtually all focusing exclusively on between-person differences, key foci of this objective were (a) to examine associations of HbO mean and variability with cognitive function at both the between- and within-person levels, (b) to document whether the patterns were similar in magnitude and direction of effect (an empirical question given the fact that between- and within-person findings need not be identical), and (c) to establish benchmark patterns for both the between- and within-person effects, representing patterns for unconfounded sources of variance, to be replicated in subsequent studies.

Finally, a related research objective pertains to understanding whether the complementary operationalizations (mean and standard deviation) of cortical hemoglobin concentrations were differentially modulated as a function of cognitive load (control versus interference conditions) on the MSIT task. Neural variability remains poorly understood for higher-order cognitive measures, such as executive function, and the modulation of neural variability has not been explored within persons. Consistent with some interpretations offered in the functional literature for the impact of load modulation on mean neural activations,^2^^,^^13^^,^^21^ we hypothesized that the more demanding interference condition would result in greater recruitment of neural tissue,^2^ but that the patterns would differ across operationalizations of the hemodynamic response as well by behavioral metric (accuracy versus latency) and cortical region.

## 2 Method

### 2.1 Participants

This study was approved by the University of Victoria Human Research Ethics Board and was conducted in accordance with institutional guidelines. Data were collected from a narrow-age cohort 71- to 81-years of age (m=75.88 and SD=3.28) of 25 older adults (13 females and 12 males), 92% of whom were right-handed. Exclusionary criteria included self-report of (a) a physician-diagnosed major medical illness with residual motor or sensory deficits (e.g., Parkinson’s disease, stroke, heart disease, dementia, cancer, and brain tumor), (b) severe sensory impairment (e.g., difficulty reading newspaper-sized print, difficulty hearing a normal spoken conversation, and difficulty writing or pressing buttons), (c) drug or alcohol abuse, (d) history of inpatient psychiatric treatment, (e) significant cognitive impairment (i.e., Mini Mental State Examination score below 24), or (f) English as a second language.

### 2.2 Measures

**Fig. 1:**
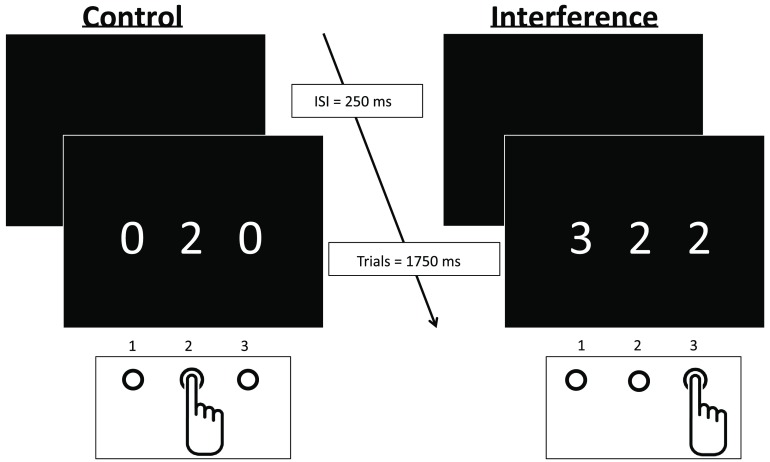
The MSIT. Participants are presented with three numbers and indicate the value of the number that is different. The value and location are congruent during control and incongruent during the interference condition.

Participants completed the MSIT, which is a computerized task of cognitive interference, and was designed to activate the cingulo-frontal-parietal cognitive attention network in subjects while undergoing functional neuroimaging.^22^^,^^23^ The task shares similarities with the Stroop, Eriksen Flanker, and Simon tasks and is suitable for the purposes of this investigation for notable reasons. Previous investigations using the MSIT have elucidated a reliable network of neuroanatomical correlates that are engaged during task performance,^22^^,^^23^ including several that are accessible by fNIRS methodology using a relatively basic array positioned over the forehead (see Fig. 1). The MSIT was designed with the assessment of neuropsychiatric populations in mind and is regularly employed in the studies of cognitive aging; however, it has yet to be used to examine variability in functional brain activity. The nature of the MSIT is such that behavioral performance can be readily yoked to neural activity, as the task demands remain relatively constant from trial to trial within a block of the experiment.

In the MSIT, participants are presented with an array of three numbers (ranging in value from 0 to 3), one of which is a different numerical value than the others. Using a serial response input device (Psychology Software Tools, Inc.) to ensure +/−1  ms timing latency, participants respond to the value of the odd target as quickly as possible while remaining accurate, across a total of 15 trials within a 30-s block. Trial durations are fixed at 2000 ms, allowing behavioral responses to be time-locked to the corresponding samples within a neuroimaging recording. Participants begin with either the control (location and value of the target are congruent) or interference condition (location and value of the target are incongruent), and complete a total of four blocks for each of the conditions, which alternate (see Fig. 1). A rest block of 20 s separates each experimental block and a baseline of at least 90 s preceded the onset of the task. A measure of interference can be derived by comparing the easier (control) to the more demanding (interference) condition, across several outcome measures of interest, including accuracy (percent correct), response time (RT), and hemodynamic response.

### 2.3 Functional Near-Infrared Spectroscopy Recording

**Fig. 2:**
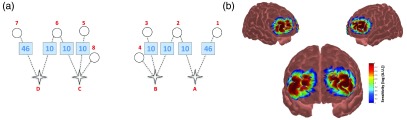
(a) The array design showing the location of the sources (star), detectors (circle), channels (dashed line), and Brodmann’s areas (squares). (b) Sensitivity profile based on the Monte Carlo forward model ($10^{6}$ photons) for the fNIRS array.

fNIRS data were recorded using a continuous-wave TechEn CW6 system (TechEn Inc., Milford, Massachusetts), with a sampling frequency of 50 Hz (corresponding to TR=20  ms) (For selected individuals (n=6), the frequency of fNIRS data acquisition was downsampled to 25 Hz. Despite this, sufficient individual samples were available to derive reliable estimates of HbO mean and variability, with analyses indicating no significant influences of these sampling differences on our estimates or pattern of results.), resulting in 100 images every 2 s. During computerized testing, the fNIRS system recorded cortical hemodynamic responses that were time-locked to events within the tasks. Participants wore custom-built fNIRS headgear consisting of an array positioned over prefrontal cortex (PFC) [Fig. 2(a)] containing 10 channels (8 at 3 cm separation, 2 at 1.5 cm separation) and were tested using wavelengths of 690 and 830 nm to index deoxyhemoglobin (HbR) and oxyhemoglobin (HbO), respectively. The array was designed to maximize the coverage of PFC, given the relevance of PFC areas during performance on the MSIT.

The optical array was positioned relative to several 10 to 20 landmarks (Fpz, Fz, F7, and F8). 3-D coordinates of scalp reference and optode locations were obtained using a Polhemus Fastrak digitizer system (Polhemus, Colchester, Vermont), to perform probabilistic spatial registration.^24^^,^^25^ Following this procedure, Montreal Neurological Institute (MNI) coordinates were generated for the midpoint of each source–detector pair (i.e., channel) for each participant, as well as the average and composite standard deviation values across the group (Table 1). Lastly, we converted the MNI coordinates to Brodmann’s areas (BA) to ascertain macroanatomical labels using Talairach Client software.^26^ The lateral-most channels in both hemispheres recorded over BA 46, with all the remaining channels recording over BA 10 [Fig. 2(b)]. The 1.5 cm short-separation channels (B4 and C8) were not of interest for the present investigation, given the inability of these channels to capture information at the cortical surface or to facilitate regression of the cardiac signal. Therefore, they were dropped from subsequent analyses. Left hemisphere channels covered inferior frontal gyrus (A1) and middle frontal gyrus (A2, B2, and B3) and right hemisphere channels covered superior frontal gyrus (C5 and C6) and middle frontal gyrus (D6 and D7). To further ascertain whether the array facilitated adequate coverage from PFC regions of interest, the probabilistic path of the light photons using the Monte Carlo forward model ($10^{6}$ photons) was simulated, to derive a sensitivity matrix^27^ [Fig. 2(b)]. This model was based on the Colin27 atlas, which specifies the absorption properties of scalp, skull, cerebral spinal fluid, gray matter, and white matter. As is evident, the array captured information that is uniformly distributed across PFC.

**Table 1:** Group average MNI coordinates for the eight-channel array.

Channel	X	Y	Z	SD
A1	−49.00	46.33	12.33	14.76
A2	−44.67	52.33	13.67	14.81
B2	−40.33	57.67	14.33	13.37
B3	−33.67	62.00	16.00	13.53
C5	32.33	64.67	16.67	19.08
C6	39.00	60.67	16.00	19.54
D6	45.67	55.33	13.67	19.89
D7	50.67	48.33	11.00	20.05

The methods undertaken as part of this study were well situated to investigate the within- and between-person associations between HbO variability and behavioral performance, as well as the modulation of neural variability by cognitive load. Although fNIRS is limited in its spatial resolution to cortical regions, the greatest age-related group differences in BOLD variability (between young and old) have emerged in relation to cortical regions.^9^ Further, the PFC in particular is linked with greater inconsistency in behavioral performance^28^ and is heavily implicated in executive functions (e.g., cognitive interference).^29^ Thus, the array used in this study is limited to the coverage of PFC, which is the area relevant to the phenomenon of interest.

### 2.4 Preprocessing

**Fig. 3:**
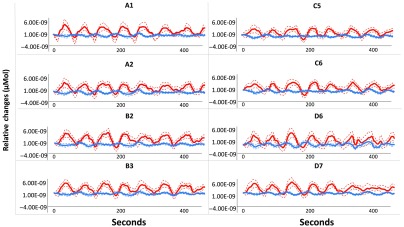
Group-averaged time-course data for the MSIT task, sampled at 50 Hz. The presence of each hemodynamic response function corresponds to a 30-s experimental block, separated by 20-s of rest.

Preprocessing of the fNIRS data was performed using Homer 2 software.^30^ After converting the raw wavelengths to optical density values, we corrected for motion using a wavelet transformation algorithm^31^ using an interquartile range of 0.1.^32^^,^^33^ Next, we applied bandpass filtering to correct for physiological noise using a high-pass filter value of 0.01 Hz and a low-pass filter value of 0.1 Hz. We then converted from optical density to hemoglobin concentrations by applying the modified Beer–Lambert law, and then exported for subsequent operationalizations. Figure 3 displays the group-averaged time-course data, sampled at 50 Hz.

### 2.5 Mean and Standard Deviation Operationalizations

Mean- and SD-based operationalizations of HbO were derived for the purpose of this study. Outliers were identified as values >3 SD from the sample mean across block estimates and were deleted pairwise, with subsequent modeling based on restricted maximum likelihood.

Mean estimates of HbO were estimated by aggregating across all samples contained within a given experimental condition. We employed a block design to index HbO within each condition. Specifically, we derived single estimates of HbO within a given block. This equated to eight segments for the MSIT (four control and four interference). Signal variance was estimated with block independent estimates, based on in-house software following the approach used by Garrett and colleagues.^3^^,^^9^^,^^10^ Percentage change from the onset value of a given block was computed for each sample, with the corresponding values subsequently averaged within a given block. Given our interest in examining the within-person time-varying covariation between HbO estimates (mean and SD) and behavioral performance (described further below), we did not normalize concatenated blocks to examine single SD estimates, condition wise. In similar fashion, the corresponding estimates for SD HbO were computed per block to facilitate estimation of the time-varying covariation models examining within-person associations between neural activity and cognitive performance.

## 3 Results

### 3.1 Behavioral Data

MSIT scores were first screened for blocks with accuracy performance <50%. Given the nature of the task and potential executive functioning difficulties experienced by the participants, there were several blocks of interference results in which participants appeared to have reversed the criteria, responding to the location of the target instead of its value. These blocks were removed from both behavioral and fNIRS analyses (36 interference blocks removed from a total of 100 across all participants). Table 2 displays the demographic and behavioral data.

**Table 2:** Descriptive statistics for the demographic and behavioral data. Data for MSIT control and interference conditions are based on blocks with at least 50% accuracy and RT data are based on correct trials only.

	Mean	SD	Min	Max
Years of education	17.40	2.77	11	22
Age	75.88	3.28	71	81
Control accuracy	97.93	3.89	85	100
Control RT	631.22	128.80	484.92	1073.85
Interference accuracy	82.13	13.49	53	100
Interference RT	1142.09	126.43	988.20	1449.75

### 3.2 Examining Within- and Between-Person Associations of Cognitive Function with HbO Mean and Variability

Hierarchical linear and nonlinear modeling (HLM) 6.08 software was used to fit linear mixed models to examine the time-varying covariation (or “coupling”) between each operationalization of hemoglobin (i.e., mean and SD) and behavioral performance (i.e., MSIT accuracy and response time), with separate models for each fNIRS channel (i.e., A1, A2, B2, B3, C5, C6, D6, and D7). In order to avoid a type II error and incorrectly rejecting a true finding, we did not correct for multiple comparisons.^34^ Rather, given that the MSIT and variability operationalizations have not been used with fNIRS in older adults, our preference was to provide a full account of the results. Given our *a priori* hypotheses regarding expected directional effects, we employed one-tailed (p<0.05) tests for specific planned comparisons. To derive distinct between- and within-person estimates of cortical hemoglobin on behavioral performance, each person-mean operationalization of hemoglobin was centered, before entering it into the model.^20^ In person-mean centering, the person mean of the time-varying predictor is subtracted from the original time-varying predictor, such that the new time-varying predictor represents variation about one’s own mean level, and thus facilitates the partitioning of HbO-cognition associations into discrete and orthogonal between- and within-person estimates. The following equations outline the analyses conducted to examine the block-to-block covariation between hemoglobin and behavioral performance (i.e., accuracy and response latency):

$${\text{Behavior}}_{ij}=\beta_{0i+}\beta_{1i}({\text{block}}_{ij})+\beta_{2i}({\text{hemoglobin}}_{ij}-{\text{hemoglobin}}_{.j})+e_{ij}\;(\text{level-}1)\;\beta_{0i}=\gamma_{00}+\gamma_{01}({\text{hemoglobin}}_{.j})+u_{0i}\;(\text{level-}2)\;\beta_{1i}=\gamma_{10}+u_{1i}\;\beta_{2i}=\gamma_{20}+u_{2i}$$

where behavior represents the outcome measures of accuracy or response latency for person j and block i. Within-person variance is reflected in the level-1 residuals $\mathrm{Var}(e_{ij})$, associated with within-person variability block to block. Between-person variance is reflected in the level-2 residuals, $\mathrm{Var}(U_{0j})$ and indicates the amount of variability in behavior that exists between-persons. The parameter estimate for $\gamma_{10}$ represents the between-person effect of block on the cognitive outcomes (MSIT accuracy and RT), with parameter $\gamma_{00}$ (the fixed intercept) reflecting the between-person average of the cognitive outcome for values of 0 on all predictors (e.g., the very first block for the person-mean-centered values of SD HbO). The parameter estimate for $\gamma_{01}$ represents the test of between-person differences in the predictors (mean and SD HbO) on the cognitive outcomes. Specifically, for every unit increase in the person mean of mean or SD HbO, the mean of the cognitive outcome goes up (or down) by the value of the $\gamma_{01}$ parameter estimate. Essentially, this parameter reflects the extent to which, for every unit increase in the person mean of HbO (based on either mean or SD computation), the mean of the behavioral performance outcome variable increases or decreases. In contrast, the $\gamma_{20}$ parameter estimate represents the test of coupled within-person associations between the predictor and the outcome. Within any given individual, within-person coupling tests whether on occasions when mean or SD HbO is higher, the corresponding estimates of cognitive performance are lower (or higher). Essentially, this parameter reflects the extent to which deviations from an individual’s average amount of HbO across the eight experimental blocks (based on either mean or SD computation) are associated with differences in the same individual’s behavioral performance. The results for the between- versus within-person analyses do not have to be identical and can conceivably differ in magnitude of effect, direction of effect, or both.^17^^,^^18^^,^^20^

**HbO mean:** The within- and between-person associations of HbO mean on variation in behavioral performance were tested separately for each behavioral metric and fNIRS channel. Table 3 summarizes the effects. At the between-person level, greater HbO concentration in the control condition was associated with less accurate performance in two adjacent left hemisphere channels ($A_{1}$: $\gamma_{01}=-0.022$, p=0.029; $A_{2}$: $\gamma_{01}=-0.018$, p=0.012). At the within-person level, greater HbO concentration in the control condition was associated with faster performance in one right hemisphere channel ($D_{6}$: $\gamma_{20}=-23.14$, p=0.019, $\text{pseudo}\;R^{2}=0.137$), such that on blocks when participants recruited more HbO relative to their own mean, they tended to perform faster (23.14 ms faster for every μMol increase in mean HbO, relative to a given person’s average). Of the total within-person variation in response time, 13.7% was accounted for by changes in HbO concentration, with the pseudo-$R^{2}$ estimate based upon the Snijders and Bosker computation.^35^^,^^36^ Greater HbO concentration in the interference condition was associated with less accurate performance in both left and right hemisphere channels ($A_{1}$: $\gamma_{01}=-0.104$, p=0.015; $D_{6}$: $\gamma_{01}=-0.060$, p=0.074) and with slower performance in two left hemisphere channels ($A_{1}$: $\gamma_{01}=68.46$, p=0.063; $B_{3}$: $\gamma_{01}=59.55$, p=0.085) at the between-person level. At the within-person level, greater HbO concentration was associated with less accurate performance in one right hemisphere channel ($D_{7}$: $\gamma_{20}=-0.060$, p=0.063, $\text{pseudo-}R^{2}=0.049$), such that on occasions when participants recruited more HbO relative to their own mean, they tended to perform less accurately (0.06% less accurate for every μMol increase in mean HbO, relative to a given person’s average). Of the total within-person variation in accuracy, 4.9% was accounted for by changes in HbO concentration.

**Table 3:** Two-level multilevel models examining between- and within-person associations for mean HbO with cognitive performance. Coefficients are reported for the between- ($\gamma_{01}$) and within-subject ($\gamma_{20}$) slope estimates. MSIT, multisource interference task; BS, between-subject; WS, within-subject; BA, Brodmann’s areas.

	MSIT accuracy		MSIT response time	
Control	HbO-BS ($\gamma_{01}$)	HbO-WS ($\gamma_{20}$)	HbO-BS ($\gamma_{01}$)	HbO-WS ($\gamma_{20}$)
BA 46 (A1)	−0.022**	0.005	−14.68	−11.97
BA 10 (A2)	−0.018**	−0.002	−2.09	−0.07
BA 10 (B2)	−0.006	−0.024	−2.99	0.96
BA 10 (B3)	−0.007	−0.008	7.43	−0.32
BA 10 (C5)	−0.005	−0.002	−8.99	−9.95
BA 10 (C6)	−0.002	−0.003	13.78	−11.20
BA 10 (D6)	−0.008	−0.002	−7.88	−23.14**
BA 46 (D7)	−0.007	−0.004	−21.00	−9.95
Interference	HbO-BS ($\gamma_{01}$)	HbO-WS ($\gamma_{20}$)	HbO-BS ($\gamma_{01}$)	HbO-WS ($\gamma_{20}$)
BA 46 (A1)	−0.104**	0.016	68.46*	−9.23
BA 10 (A2)	−0.024	−0.008	21.15	22.09
BA 10 (B2)	−0.003	−0.016	34.27	21.77
BA 10 (B3)	−0.025	−0.015	59.55*	12.22
BA 10 (C5)	0.040	−0.020	34.20	41.95
BA 10 (C6)	0.021	−0.022	44.90	14.52
BA 10 (D6)	−0.060**	0.044	22.41	90.48
BA 46 (D7)	0.014	−0.060*	17.41	97.83

* p<0.05, one-tailed.

** p<0.05, two-tailed.

**HbO SD:** The within- and between-person associations of HbO SD on variation in behavioral performance were tested separately for each behavioral metric and fNIRS channel.

Table 4 summarizes the effects. At the between-person level, greater HbO variability in the control condition was associated with slower performance in two adjacent left hemisphere channels ($A_{1}$: $\gamma_{01}=0.66$, p=0.041; $A_{2}$: $\gamma_{01}=0.39$, p=0.095). At the within-person level, greater HbO variability in the control condition was associated with faster performance in one left hemisphere channel ($A_{2}$: $\gamma_{20}=-0.09$, p=0.089, $\text{pseudo-}R^{2}=0.063$), such that on blocks when participants were more variable in HbO relative to their own average variability, they tended to perform faster (0.09 ms faster for every μMol increase in SD HbO, relative to a given person’s average). Of the total within-person variation in response time, 6.3% was accounted for by changes in HbO variability. We also found that greater HbO variability in the interference condition was associated with more accurate performance in one right hemisphere channel ($D_{6}$: $\gamma_{20}=29.0^{-4}$, p=0.087, $\text{pseudo-}R^{2}=0.243$), such that on blocks when participants were more variable in HbO relative to their own mean, they tended to perform more accurately (0.003% more accurate for every μMol increase in SD HbO, relative to a given person’s average). Of the total within-person variation in accuracy, 24.3% was accounted for by changes in HbO variability.

**Table 4:** Two-level multilevel models examining between- and within-person associations for SD HbO with cognitive performance. Coefficients are reported for the between- ($\gamma_{01}$) and within-subject ($\gamma_{20}$) slope estimates. MSIT, multisource interference task; BS, between-subject; WS, within-subject; BA, Brodmann’s areas.

	MSIT accuracy		MSIT response time	
Control	HbO-BS ($\gamma_{01}$)	HbO-WS ($\gamma_{20}$)	HbO-BS ($\gamma_{01}$)	HbO-WS ($\gamma_{20}$)
BA 46 (A1)	$1.42^{-4}$	$-0.13^{-4}$	0.66**	0.02
BA 10 (A2)	$0.70^{-4}$	$0.18^{-4}$	0.39*	−0.09*
BA 10 (B2)	$0.64^{-4}$	$1.85^{-4}$	−0.14	−0.00
BA 10 (B3)	$-0.35^{-4}$	$0.08^{-4}$	0.23	−0.05
BA 10 (C5)	$0.06^{-4}$	$0.38^{-4}$	0.05	−0.08
BA 10 (C6)	$-0.44^{-4}$	$0.04^{-4}$	−0.02	0.06
BA 10 (D6)	$0.05^{-4}$	$0.42^{-4}$	0.12	0.02
BA 46 (D7)	$0.55^{-4}$	$0.67^{-4}$	0.24	0.15
Interference	HbO-BS ($\gamma_{01}$)	HbO-WS ($\gamma_{20}$)	HbO-BS ($\gamma_{01}$)	HbO-WS ($\gamma_{20}$)
BA 46 (A1)	$2.81^{-4}$	$-0.45^{-4}$	−0.02	0.03
BA 10 (A2)	$-0.26^{-4}$	$-1.04^{-4}$	0.28	−0.15
BA 10 (B2)	$2.11^{-4}$	$-1.92^{-4}$	−0.12	−0.11
BA 10 (B3)	$0.95^{-4}$	$1.13^{-4}$	−0.16	−0.16
BA 10 (C5)	$-5.53^{-4}$	$-0.26^{-4}$	0.21	−0.15
BA 10 (C6)	$-2.56^{-4}$	$0.11^{-4}$	0.01	0.05
BA 10 (D6)	$0.76^{-4}$	$29.0^{-4}$*	−0.05	0.36
BA 46 (D7)	$-9.47^{-4}$	$-0.59^{-4}$	0.08	0.23

* p<0.05, one-tailed.

** p<0.05, two-tailed.

## 4 Discussion

This investigation sought to examine complementary operationalizations of the cortical hemodynamic response for older adults completing a measure of executive functioning. Neuroimaging data have historically been examined through central tendency computations, in order to derive what have been perceived as more stable estimates based upon block averaging of neural activity. Recent work has revisited previous assertions that neural variability is not merely noise^9^^,^^10^ and has shown that the variability inherent in neural activity conveys functional significance, including associations with brain maturation,^5^^–^^7^ brain senescence,^3^^,^^9^^,^^10^ behavioral performance,^3^^,^^6^^,^^8^^,^^10^^–^^12^ and better recovery from TBI.^14^ The variability literature on cerebral oxygenation has been largely restricted to fMRI methodology, which is limited by temporal sampling resolution. Thus, more densely sampled profiles of variability for cerebral oxygenation remain virtually unexplored. Another impetus for the present investigation was to examine neural variability using fNIRS and to exploit its comparatively faster sampling frequency to derive variability estimates based upon a greater number of samples that may be more statistically reliable. Further, the ecological advantages of fNIRS (e.g., low cost, portability, and noninvasiveness) may be particularly advantageous for studying variability outside of highly controlled laboratory settings. Finally, although evidence continues to mount in favor of the functional significance of neural variability, it is less clear whether these effects are driven by within- or between-person factors. As emphasized earlier, effects at the between- versus within-person level of analysis may systematically differ due to unaccounted for confounds (e.g., age group differences in performance and individual differences in practice) or due to fundamental differences in the theoretical constructs indexed at each level of analysis (e.g., between-person differences in neural variability may reflect systemic differences in level of central nervous system function, whereas within-person fluctuations in neural activations may reflect more transient influences including stress or momentary lapses of attention). To the extent that variation between- versus within-persons reflects distinct underlying sources, the observed patterns for these distinct levels of analysis may differ in magnitude or direction of effect, or both.^20^ Thus, it is both conceptually and statistically feasible that as a predictor, neural variability may account for variance at the between- but not the within-person level (i.e., that it may be associated with superior behavioral performance, but be driven by between-person factors). Higher-order cognitive tasks may be more likely to exhibit practice effects relative to other cognitive constructs of interest, further underscoring the importance of separating within- and between-person sources of variance. Among the few fNIRS studies to examine variability, large between-group differences have been reported;^37^ however, no investigations of variability within-persons have been reported to our knowledge.

Using fNIRS, two operationalizations of HbO were derived, based on central tendency (mean) and variability (SD). Behavioral associations with each operationalization of HbO differed across task difficulty (control or interference), behavioral metric (accuracy or response latency), cortical area, and level of analysis (between- versus within-person). In general, greater amounts of mean HbO (i.e., higher mean activation) were associated with less accurate and slower response in both MSIT conditions, in lateral regions of PFC (including DLPFC). Greater amounts of mean HbO were associated with faster performance in the easier control condition, but with slower performance in the more challenging interference condition. One interpretation of this finding is that recruiting additional neural tissue may have been beneficial as the task remained relatively easy, but that as it became more challenging, additional recruitment of neural tissue was no longer able to compensate for the increased task demands.^2^ Greater variability in HbO at the between-person level showed some associations to slower performance, especially in the easier condition. At the within-person level, however, greater variability in HbO showed associations to greater accuracy and faster performance in both conditions, with these effects occurring predominantly in lateral areas of PFC. These within-person patterns replicate previous fMRI findings linking BOLD variability to faster and more accurate cognitive performance.^20^

On balance, these results are in keeping with fMRI findings that mean and variability in the HbO signal confer complementary sources of information.^9^ Although greater mean was associated with accuracy more so than response time, variability showed a trend for the opposite pattern and showed more reliable associations to response time. With regard to measurement reliability of rapidly changing internal dynamics, response time metrics may afford more reliable estimates relative to accuracy, given the sensitivity of the scales associated with each metric.^38^^–^^40^ Accordingly, the greater proportion of HbO variability associations with response time suggests that neural variability may be effectively capturing moment-to-moment changes in internal dynamics. Of additional note is that these effects were observed at both between- and within-person levels. Although virtually all studies have tested neural variability–cognition associations at the population (group) rather than at individual level, the most theoretically-empirically matched test of the hypothesis (i.e., that on occasions when neural variability is higher, corresponding estimates of cognitive function are also higher) should be demonstrated within-persons.^41^ For the more conservative within-person test, greater HbO variability tended to couple with better behavioral performance, in keeping with previous claims that greater variability is adaptive.^1^^,^^3^^–^^15^ In contrast, the findings linking greater between-person variability to poorer cognitive function may be artifactual based upon population mean confounds, such as age differences.^18^ Although the coverage of cortical regions is limited by both methodology (fNIRS) and design (array covering regions of PFC only), these preliminary findings are consistent with previous results demonstrating that mean and variability are spatially distinct and are orthogonal in nature.^9^

### 4.1 Limitations and Future Directions

The observed effects that have been reported in this study are limited due to sample size constraints and duration of task (i.e., number of blocks), resulting in decreased power. To ascertain fully the extent to which each operationalization of HbO is driven by within- or between-person effects, a greater sample size is required; ideally, one in which the incidence of a potential cognitive impairment is apparent (e.g., mild cognitive impairment). Similarly, additional experimental blocks would allow for greater exploration of the within-person associations between HbO and behavior. Event-related designs will also allow for more precise yoking of neural variability with behavioral performance, with an increased number of blocks increasing statistical power for the coupling analyses. The use of short-separation channels would have allowed for a more precise estimate of high-frequency physiological artifacts (e.g., Mayer waves),^42^ and future studies would benefit from this approach. In using bandpass filtering with relatively conservative thresholds, the reported variability operationalization may result in underestimating the true relationship of neural variability with behavioral performance; future research might replicate these findings employing less conservative screening criteria. As previous results have shown patterns of mean- and SD-based computations of neural activity that are spatially and inferentially distinct,^9^ the discrepancies reported here between associations of each operationalization of HbO with behavioral performance are likely to become clearer with greater statistical power. The block normalized SD computation was conducted for each channel yielding a total of eight estimates; a distinct approach from that employed in fMRI studies to date. In these studies, a whole-brain estimate across all voxels and regions is derived, which is used subsequently during inferential statistical comparisons (e.g., between young and old adults). Future investigations may seek to examine regional differences in neural variability (e.g., between frontal and parietal cortices) using expanded head coverage, as well as whole-brain SD, to facilitate comparison with fMRI reports. Given the originality of neural variability operationalizations in the fNIRS literature, replication and extension of the reported findings will be essential. Multimodal fNIRS–fMRI recording in particular would allow for an investigation of the comparability of the neural variability signal derived from the same collection.

## 5 Conclusions

Variability operationalizations of neuroimaging data are emerging as complementary metrics to conventionally employed mean-based computations. Our results demonstrate that variability in HbO recorded using fNIRS is sensitive to behavioral performance and further substantiate claims that increased neural variability is adaptive. Notably, these associations were apparent at the within-person level, suggesting that they were not driven by between-person confounds such as age differences or individual differences in practice or learning. These patterns are consistent with suppositions that increased neural variability connotes moment-to-moment fluctuations indicative of a nimble, responsive, and dynamic system.^3^^,^^43^ Mean and variability operationalizations of HbO may be sensitive to different metrics of behavioral performance (accuracy or response time), with variability showing greater sensitivity for moment-to-moment changes in rapidly changing internal dynamics. Additional work is needed to further examine the associations between alternative operationalizations of HbO from tasks of varying complexity (e.g., examining the modulation of the HbO signal in tasks that vary in complexity and difficulty). The relatively high temporal sampling frequency of the current fNIRS systems places the methodology in good standing for such future work, as it facilitates deriving variability estimates that are relatively precise and reliable. Further, this line of research may represent a next frontier in facilitating our understanding of the complex interrelations among brain function, cognition, and age.
